# Exendin-4 Pretreatment Attenuates Kainic Acid-Induced Hippocampal Neuronal Death

**DOI:** 10.3390/cells10102527

**Published:** 2021-09-24

**Authors:** Yu-Jeong Ahn, Hyun-Joo Shin, Eun-Ae Jeong, Hyeong-Seok An, Jong-Youl Lee, Hye-Min Jang, Kyung-Eun Kim, Jaewoong Lee, Meong-Cheol Shin, Gu-Seob Roh

**Affiliations:** 1 Bio Anti-Aging Medical Research Center, Department of Anatomy and Convergence Medical Science, Institute of Health Sciences, College of Medicine, Gyeongsang National University, Jinju 52727, Korea; ahnujung@naver.com (Y.-J.A.); k4900@hanmail.net (H.-J.S.); jeasky44@naver.com (E.-A.J.); gudtjr5287@hanmail.net (H.-S.A.); jyv7874v@naver.com (J.-Y.L.); gpals759@naver.com (H.-M.J.); kke-jws@hanmail.net (K.-E.K.); woongs1111@gmail.com (J.L.); 2 Research Institute of Pharmaceutical Sciences, College of Pharmacy, Gyeongsang National University, Jinju 52828, Korea; shinmc@gnu.ac.kr

**Keywords:** exendin-4, neuronal death, hippocampus, kainic acid, seizures

## Abstract

Exendin-4 (Ex-4) is a glucagon-like peptide-1 receptor (GLP-1R) agonist that protects against brain injury. However, little is known about the effect of Ex-4 on kainic acid (KA)-induced seizures and hippocampal cell death. Therefore, this study evaluated the neuroprotective effects of Ex-4 pretreatment in a mouse model of KA-induced seizures. Three days before KA treatment, mice were intraperitoneally injected with Ex-4. We found that Ex-4 pretreatment reversed KA-induced reduction of GLP-1R expression in the hippocampus and attenuated KA-induced seizure score, hippocampal neuronal death, and neuroinflammation. Ex-4 pretreatment also dramatically reduced hippocampal lipocalin-2 protein in KA-treated mice. Furthermore, immunohistochemical studies showed that Ex-4 pretreatment significantly alleviated blood–brain barrier leakage. Finally, Ex-4 pretreatment stimulated hippocampal expression of phosphorylated cyclic adenosine monophosphate (cAMP) response element-binding protein (p-CREB), a known target of GLP-1/GLP-1R signaling. These findings indicate that Ex-4 pretreatment may protect against KA-induced neuronal damage by regulating GLP-1R/CREB-mediated signaling pathways.

## 1. Introduction

The kainic acid (KA) mouse model of seizures and epilepsy has been proven to be an important system for understanding seizure pathology and developing therapeutic agents. KA-induced seizures are characterized by neuronal cell death, neuroinflammation, blood–brain barrier (BBB) leakage, and oxidative stress [1,2,3]. KA treatment also increases metabolic demands, resulting in heightened glucose utilization, neuronal damage, and oxidative stress [4,5]. Cerebral blood flow increases in the hippocampus to compensate for local oxygen and glucose demands under KA-induced excitotoxicity [6,7]. In addition, our previous study showed that high-fat diet feeding promotes KA-induced hippocampal neuronal loss [8]. Whether therapeutic agents that alleviate excitotoxicity-induced energy demands affect hippocampal neuronal death is unknown.

Glucagon-like peptide-1 (GLP-1), an incretin hormone, reduces blood glucose levels by stimulating insulin secretion and suppressing glucagon release [9]. In particular, native GLP-1 and exendin-4 (Ex-4) cross the BBB and these peptides are capable of directly penetrating and activating central nervous system centers after exogenous administration [10,11]. Several studies have shown that activation of GLP-1/GLP-1 receptor (GLP-1R) signaling leads to protection against memory loss, neuroinflammation, and neurotoxicity [12,13,14,15]. Additionally, GLP-1R-mediated phosphorylation of cyclic adenosine monophosphate (cAMP) response element-binding protein (p-CREB) plays a role in neuronal development, synaptic plasticity, and memory formation in neurons [16]. GLP-1R/CREB signaling also induces expression of neuroprotective genes such as brain-derived neurotrophic factor and Bcl-2 [17]. The neuroprotective role of agonist of GLP-1R and its precise mechanisms have been studied in neuropathological disorders such as Alzheimer’s disease, Parkinson’s disease, vascular brain injury, epilepsy, and so on [9,13,15,18,19].

Therapies that target GLP-1/GLP-1R signaling may have neuroprotective effects in the KA-induced seizure mouse model. Ex-4 is a GLP-1R agonist that shares 53% amino acid homology with GLP-1. Ex-4 mimics the activity of GLP-1R but has a longer half-life due to its resistance to enzyme dipeptidyl-peptidase-4 (DPP-4) degradation [16]. Already used as a treatment for type 2 diabetes mellitus, Ex-4 has been shown by several studies to exert neuroprotective effects in brain injury models, such as subarachnoid hemorrhage, hyperthermia-induced seizures, and cerebral ischemia [9,12,16,20]. Although GLP-1 or Ex-4 protects primary hippocampal neurons from glutamate-induced neuronal death [21], the precise mechanism by which Ex-4 prevents hippocampal neuronal cell death in KA-induced seizures is not fully understood.

In the current study, we explored the effects of Ex-4 pretreatment on hippocampal neuronal death, neuroinflammation, and BBB breakdown in KA-induced seizure mice. Additionally, we determined whether Ex-4 pretreatment induced GLP-1R/CREB signaling in response to KA treatment in mice. Our findings suggest that activation of GLP-1R by Ex-4 pretreatment protected against hippocampal damage via upregulation of the p-CREB signaling pathway.

## 2. Materials and Methods

### 2.1. Animals

Male ICR mice (5 weeks old, *n* = 63) were obtained from KOATECH (Pyeongtaek, Korea) and maintained at Gyeongsang National University’s animal facility (GNU). All animal research at GNU was approved by the Institutional Board of Research (GNU-190701-M0033). For laboratory animal care, we followed the National Institutes of Health recommendations. Mice were housed in a 12 h light/dark cycle.

### 2.2. Ex-4-Albumin Binding Domain (ABD) Protein Expression and Purification

The pET28a-small ubiquitin-like modifier (SUMO)-Ex-4-ABD vector was constructed by double digestion of the Ex-4-ABD coding gene (336 bp, GenScript) with Nde1 and EcoRI followed by ligation into linearized pET28a-SUMO. A colony of pET28a-SUMO-Ex-4-ABD transformed BL21 (DE3) E. coli cells was picked and used to inoculate 50 mL Luria-Bertani (LB) broth containing 80 µg/mL kanamycin. This starter culture was shaken at 250 rpm and incubated at 37 °C overnight. After incubation, the starter culture was added to 1 L fresh LB broth with 80 µg/mL kanamycin and then incubated as described above. When the absorbance at 600 nm (OD600) reached 1.0, isopropyl β-d-1-thiogalactopyranoside (IPTG) was added as the inducer (at a final concentration of 0.5 mM), and the batch culture was incubated for an additional 4 h. After incubation, cells were harvested by centrifugation and then resuspended in 60 mL of 20 mM phosphate-buffered saline (PBS) (pH 7) containing 300 mM NaCl, 1% leupeptin, and 1% soybean protease inhibitor (Sigma-Aldrich, St. Louis, MO, USA). After sonication and centrifugation of the cell suspension, the supernatant fraction containing soluble SUMO-Ex-4-ABD was placed onto Talon^®^ metal affinity resins (Clontech, Mountain View, CA, USA). After washing with PBS, Ex-4-ABD was eluted with elution buffer (20 mM PBS, 300 mM NaCl, 300 mM imidazole, pH 7). The production yield of Ex-4-ABD, as determined by a bicinchoninic acid (BCA) protein assay, was 8.9 mg/L culture, and the purity was above 80% as calculated by densitometry analysis using ImageJ (Version 1.52a, NIH, Bethesda, MD, USA).

### 2.3. Drug Treatment and Seizure Mouse Model

Experiment 1: Experimental mice (KA, *n* = 15) were treated with an intraperitoneal injection of KA (30 mg/kg; Abcam, Cambridge, MA, USA). Control mice (CTL, *n* = 4) were intraperitoneally injected with an equivalent volume of 0.9% normal saline. Mice in the experimental group were sacrificed at 2, 6, or 24 h after injection (*n* = 4 mice per group).

Experiment 2: To assess the neuroprotective effects of Ex-4 pretreatment in mice with KA-induced seizures, Ex-4 (200 nmol/kg) was dissolved in 0.9% normal saline and intraperitoneally injected 72 h before KA injection. We previously determined the neuroprotective concentration of Ex-4 in diabetic mouse models [22,23]. The animals were divided into four groups at random: control mice (CTL, *n* = 10), KA-induced seizure mice (KA, *n* = 12), KA and Ex-4-treated mice (KA + Ex-4, *n* = 12), and Ex-4-treated (only) mice (*n* = 10). Seizure activity was evaluated using a 6-point seizure scale during the 2 h observation period after KA administration as previously described [24]. Seizures were graded on a six-point scale based on their severity (I to VI); I = huddled in a corner, motionless and gazing, II = body extends out, tail becomes straight and stiff, ears are pushed back, eyes bulge, III =Rears into a sitting position, forepaws resting on bell, and bobbles his head repeatedly, IV = tonic for raising and lowering Jumping clonus, running clonus, and clonic convulsions with periods of absolute stillness, V = continuous level IV seizures, VI = body in clonus, limbs no longer used to maintain posture, frequently a sign of impending death. All mice were sacrificed at 24 h after KA treatment.

### 2.4. Enzyme-Linked Immunosorbent Assay (ELISA)

To measure Ex-4 in serum and hippocampal tissues, mice were anesthetized with Zoletil (5 mg/kg; Virbac Laboratories, Carros, France) 24 h after KA administration. Blood samples were obtained by cardiac puncture of the left ventricle and then centrifuged. Hippocampi were removed from the brain. Ex-4 levels in both serum and hippocampal tissues were quantified using an ELISA kit (MyBioSource, San Diego, CA, USA) according to the manufacturer’s instructions (*n* = 8 mice per group).

### 2.5. Tissue Preparation and Cresyl Violet Staining

Mice were perfused transcardially with 0.9% normal saline followed by 4% paraformaldehyde (PFA) in 0.1 M PBS. Then, the extracted brains were post-fixed in PFA for 6 h and sequentially immersed in 0.1 M PBS containing 15% sucrose and 0.1 M PBS containing 30% sucrose at 4 °C until submerged. Brains were cut into 40-µm thick coronal sections. The sections were stained with Cresyl violet (Sigma-Aldrich) and imaged with a BX53 light microscope (Olympus, Tokyo, Japan).

### 2.6. Terminal Deoxynucleotidyl Transferase dUTP Nick End Labeling (TUNEL) Assay

TUNEL was conducted on frozen tissue sections according to the manufacturer’s protocol using an in situ cell death detection kit (Roche Molecular Biochemicals, Mannheim, Germany). The sections were examined using a BX51 DSU microscope (Olympus), and digital pictures were taken.

### 2.7. Western Blot Analysis

For total protein isolation, frozen hippocampi were homogenized in tissue protein extraction reagent (T-per) lysis buffer (Thermo Fisher Scientific, Waltham, MA, USA). Homogenized hippocampi were incubated on ice for 20 min and sonicated three times. Samples were centrifuged for 30 min at 12,000 rpm at 4 °C, and supernatants were transferred to clean vials. The cytosolic and mitochondrial fractions were obtained using a mitochondria isolation kit for tissue (Thermo Fisher Scientific). Protein concentrations were determined using a Bio-Rad protein assay (Hercules, CA, USA), and samples were stored at -80 °C until use. Total proteins (10–20 μg) were separated by SDS-PAGE on 8–12% acrylamide gels and transferred onto membranes. The membranes were probed with primary antibodies (Supplementary Table S1). β-actin, VDAC-1 and IgG were used as internal controls to normalize protein levels. Band densitometry was measured using the Multi-Gauge V 3.0 image analysis program (Fujifilm, Tokyo, Japan).

### 2.8. Proximity Ligation Assay (PLA)

To determine whether two molecules were in sufficient proximity to interact, the Duolink^®^ in situ PLA (OLINK Bioscience, Uppsala, Sweden) was performed according to the manufacturer’s manuals. Anti-rabbit MINUS (first PLA probe) detected rabbit Ex-4 antibody, whereas anti-mouse PLUS (second PLA probe) detected mouse GLP-1R antibody (Supplementary Table S1). After performing the assay, sections were stained with DAPI (1:10,000, Invitrogen, Carlsbad, CA, USA), washed, and coverslipped using mounting medium. Images of sections were captured by BX51 DSU microscope (Olympus).

### 2.9. Double Immunofluorescence

Brain sections were incubated with primary antibodies (Supplementary Table S1). For double immunofluorescence staining, free-floating brain sections were incubated with rabbit anti-GLP-1R and mouse anti-NeuN or mouse anti- glial fibrillary acidic protein (GFAP); goat anti- lipocalin-2 (LCN2) and mouse anti-GFAP; rat anti-zonula occludens-1 (ZO-1); and goat anti-aquaporin-4 (AQP4) and rabbit anti-albumin at 4 °C. Sections were then incubated with donkey secondary antibodies conjugated with Alexa Fluor 488 and 594 (Invitrogen). Sections were counterstained with 4′,6-diamidino-2-phenylindole (DAPI, 1:10,000, Invitrogen), washed, and coverslipped using a mounting medium. Images of the sections were visualized under BX51 DSU microscope (Olympus).

### 2.10. Immunohistochemistry

Brain sections were incubated with rabbit anti-ionized calcium-binding adapter molecule-1 (Iba-1). After 14 h at 4 °C, slices were washed and treated with a biotinylated secondary antibody (Thermo Fisher Scientific) for 1 h at room temperature. Brain sections were processed with an avidin-biotin complex (ABC) solution (Vector Laboratories, Burlingame, CA, USA) and a 3,3′-Diaminobenzidine (DAB) kit (Sigma-Aldrich), mounted on gelatin-coated slides and coverslipped with Permount (Sigma-Aldrich). The stained sections were visualized with BX53 light microscope (Olympus), and digital images were captured.

### 2.11. Quantification of Histological Data

According to mouse atlas (the mouse brain in stereotaxic coordinates, Academic Press), we selected two coronal brain sections per mouse brain (*n* = 3–4 mice per group) between Bregma level −1.58 mm to −2.18 mm. For each treatment group, TUNEL-positive cells, Duolink-stained signals, and immunostained positive cells were counted in 8–10 CA3 regions (200 × 200 µm^2^) using automatic quantitative component image analysis software (I-solution^®^, IMT i-solution Inc., Burnaby, BC, Canada). For the quantification of albumin in extravascular space, we counted albumin-positive cells, which were detected outside AQP4-positive vessels in the damaged CA3 regions caused by KA using automatic image analysis software (I-solution^®^).

### 2.12. Statistical Analysis

All statistical analyses were performed with PRISM 7 (GraphPad Software Inc., San Diego, CA, USA). Statistical differences were determined by a one-way ANOVA, followed by Tukey’s test as a post hoc analysis. In addition, two-group differences were determined by Student t-tests. The mean SEM was used to represent all values in graphs. A *p*-value of 0.05 was used to determine statistical significance.

## 3. Results

### 3.1. KA Treatment Affects the Hippocampal Expression of Heme Oxygenase-1 (HO-1) and GLP-1R in Mice

**Figure 1:**
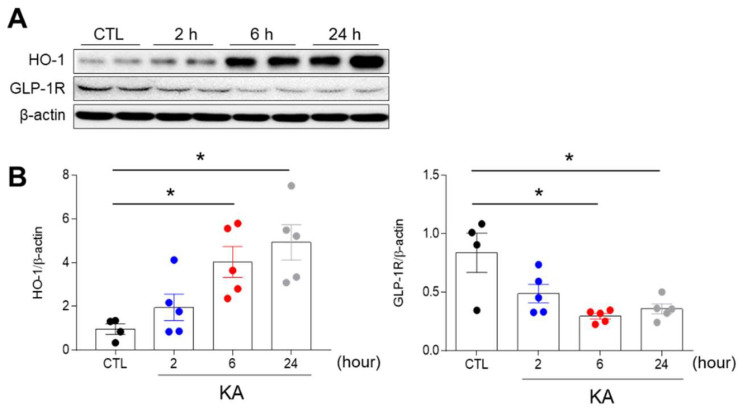
Effects of KA treatment on hippocampal HO-1 and GLP-1R expressions in KA-treated mice. (**A**) Western blot analysis of hippocampal HO-1 and GLP-1R protein expressions in mice at 2, 6, and 24 h after KA treatment. (**B**) Quantitative expressions of HO-1 and GLP-1R protein from Western blot analysis (*n* = 4–5 mice per group). Densitometric values for HO-1 and GLP-1R were normalized to β–actin levels. Data are presented as mean ± standard error of the mean (SEM). * *p* < 0.05 vs. control (CTL). The one-way analysis of variance (ANOVA) was used to detect any statistically significant differences time-dependently (**B**). HO-1/β–actin; *p* values = 0.0273 (6 h), 0.0044 (24 h), F (3, 15) = 7.478, GLP-1R/β–actin; *p* values = 0.0026 (6 h), 0.0071 (24 h), F (3, 15) = 7.446.

We first investigated the hippocampal expression of HO-1, an intrinsic factor against oxidative stress in hippocampal neurons, at the same time points after KA injection [1,25]. HO-1 was increased 6 h after KA treatment (Figure 1). GLP-1R expression plays an important role in modulating the stress response in the brain [26]. To determine the change in GLP-1R expression induced by KA, we investigated hippocampal GLP-1R expressions in mice 2, 6, and 24 h after KA injection (Figure 1). The hippocampal GLP-1R expression was dramatically reduced 6 h after KA treatment. These findings indicate that KA-induced oxidative stress could reduce hippocampal GLP-1R protein.

### 3.2. Ex-4 Pretreatment Reduces KA-Induced Seizure Activity and Hippocampal Cell Death in Mice

**Figure 2:**
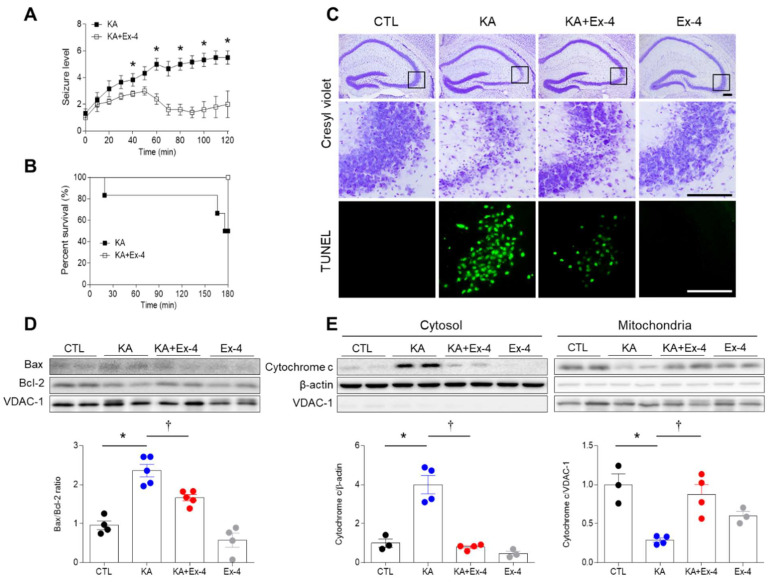
Effects of Ex-4 pretreatment on seizure activity and hippocampal cell death in KA-treated mice. (**A**) Seizure activity was measured during the two-hour monitoring period following KA injection. (**B**) The percentage of survival in KA and KA + Ex-4 group are shown (*n* = 8–10 mice per group). (**C**) Representative images of Cresyl violet and TUNEL staining are shown in the hippocampal CA3 region 24 h after KA treatment. The areas in black squares in the upper panel are magnified on the middle panel. Scale bar = 200 μm, 50 μm. (**D**) Western blot analysis of hippocampal Bax and Bcl-2 expression. Densitometric values were normalized to the Bax/Bcl-2 ratio in the CTL group (*n* = 4–5 mice per group). (**E**) Western blot analysis of hippocampal cytochrome c protein expression. Mitochondria fraction and cytosol were obtained by subcellular fractionation. Densitometric values for cytosolic and mitochondrial cytochrome c were normalized to β-actin and voltage-dependent anion-selective channel-1 (VDAC-1) levels (*n* = 3–4 mice per group). Data are presented as mean ± SEM. * *p* < 0.05 vs. CTL, † *p* < 0.05 vs. KA. The t-test was used to detect statistically significant differences between KA- and KA+Ex-4-treated mice (**A**). The one-way ANOVA was used to detect any statistically significant differences between groups (**D** and **E**). Bax/Bcl-2; *p* values = 0.0001 (KA), 0.0096 (KA+Ex-4), F (3, 14) = 33.22, Cytochrome c/β–actin; *p* values = 0.0002 (KA), 0.0001 (KA+Ex-4), F (3, 10) = 33.01, Cytochrome c/VDAC-1; *p* values = 0.0024 (KA), 0.0054 (KA+Ex-4), F (3, 10) = 10.63.

Given that KA treatment led to decreased GLP-1R expression, we evaluated whether pretreatment with Ex-4, a GLP-1R agonist, would attenuate other effects induced by KA. For 2 h after KA treatment, we evaluated the anticonvulsant effects of Ex-4 by monitoring behavioral seizure activity and percent survival. Ex-4 pretreatment did not reach seizure scores greater than grade 4 in KA + Ex-4-treated mice compared to KA-treated mice (Figure 2A). Furthermore, KA + Ex-4-treated mice exhibited a higher survival rate than those given KA alone (Figure 2B). Cresyl violet staining revealed that KA-treated mice exhibited substantial cell loss in the hippocampal CA3 region (Figure 2C). However, Ex-4 pretreatment in the KA + Ex-4-treated mice reduced apoptotic cell shapes. TUNEL staining also indicated that mice treated with Ex-4 and KA had significantly fewer apoptotic cells than mice treated with KA alone (KA: 10.07 ± 1.272, KA+Ex-4: 2.624 ± 0.8731, *p* = 0.0007).

Next, we investigated the role of intrinsic apoptotic pathways in hippocampal cell death in KA-induced seizure mice. Intrinsic apoptosis, which is triggered by the release of cytochrome c from the mitochondria into the cytosol, is regulated by a balance of pro-apoptotic factors (e.g., Bax) and anti-apoptotic factors (e.g., Bcl-2). Ex-4 pretreatment downregulated the Bax/Bcl-2 ratio was induced by KA (Figure 2D). Finally, cytochrome c expression was increased in the cytosol of KA-treated mice, but this effect was significantly reduced in mice treated with both KA and Ex-4 (Figure 2E). Together, these findings suggest that Ex-4 pretreatment protects hippocampal neurons from KA-induced cell death.

### 3.3. Ex-4 Pretreatment Prevents the Inhibition of GLP-1R Protein Expression in the Hippocampus of KA-Treated Mice

**Figure 3:**
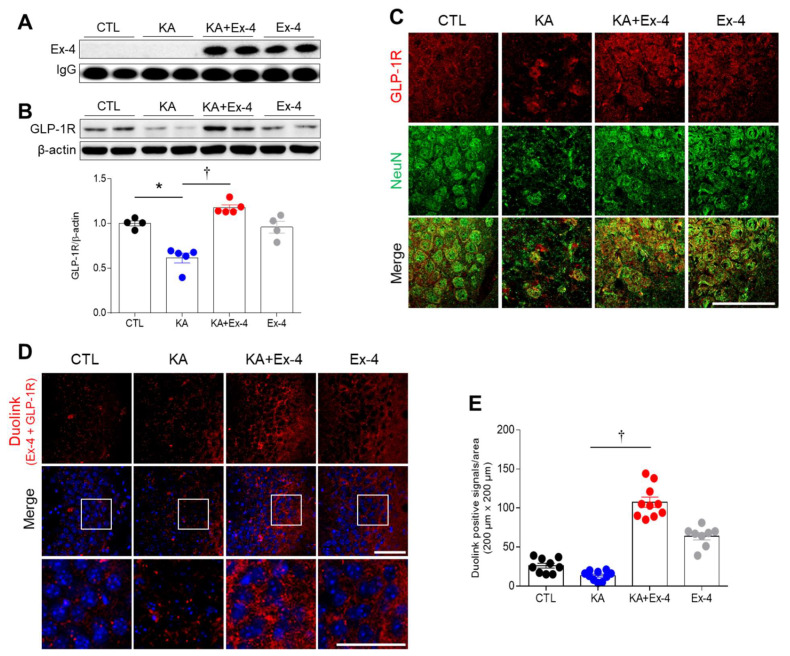
Effects of Ex-4 pretreatment on serum Ex-4 and hippocampal GLP-1R expressions in KA-treated mice. (**A**) Western blot analysis of Ex-4 levels in serum. (**B**) Western blot analysis of expressions of hippocampal GLP-1R. Densitometric values for Ex-4 and GLP-1R were normalized to immunoglobulin G (IgG) and β-actin levels (*n* = 4–5 mice per group). (**C**) Representative images of double immunofluorescence showing the co-localization GLP-1R and NeuN in the mouse hippocampus. Scale bar = 50 μm. (**D**) Representative images of Duolink assay showing the interaction of Ex-4 and GLP-1R in the hippocampal CA3 region. Nuclei were stained with 4′, 6-diamidino-2-phenylindole (DAPI). The areas in white squares in the middle panel are magnified on the lower panel. Scale bar = 50 μm. (**E**) Quantitative result of Duolink positive signals are shown in 8-10 CA3 regions (*n* = 3–4 mice per group). Data are presented as mean ± SEM. * *p* < 0.05 vs. CTL, † *p* < 0.05 vs. KA. The one-way ANOVA was used to detect any statistically significant differences between groups (**B** and **E**). GLP-1R/β–actin; *p* values = 0.0003 (KA), 0.0001 (KA + Ex-4), F (3, 14) = 26.96, E; *p* values = 0.0001 (KA + Ex-4), F (3, 33) = 96.27.

First, we assessed the circulating and hippocampal expression levels of Ex-4 across treatment groups using Western blot and ELISA analyses (Supplementary Figure S1A). In serum, Ex-4 was only detected in the KA + Ex-4 and Ex-4 treatment groups (Figure 3A; Supplementary Figure S1B). However, there was no significant difference in hippocampal expression between treated and non-treated groups in both Western blot and ELISA analyses (Supplementary Figure S1C). Next, we employed Western blot analysis to assess GLP-1R expression. KA treatment decreased protein expression of GLP-1R, whereas Ex-4 pretreatment increased expression of GLP-1R in comparison to control (Figure 3B). Double immunofluorescence showed that GLP-1R was mainly co-localized with neurons and not with GFAP, an astrocyte marker (Figure 3C; Supplementary Figure S2). Finally, we performed PLA to visualize whether Ex-4 and GLP-1R were close enough to interact in mice treated with KA and Ex-4 or KA alone (Figure 3D). Hippocampi from the KA + Ex-4-treated mice had significantly more Duolink-positive cells (Figure 3E). These findings indicate that Ex-4 pretreatment inhibited the KA-induced reduction of GLP-1R protein and was in sufficient proximity to activate GLP-1R signaling.

### 3.4. Ex-4 Pretreatment Attenuates Neuroinflammation in KA-Treated Mice

**Figure 4:**
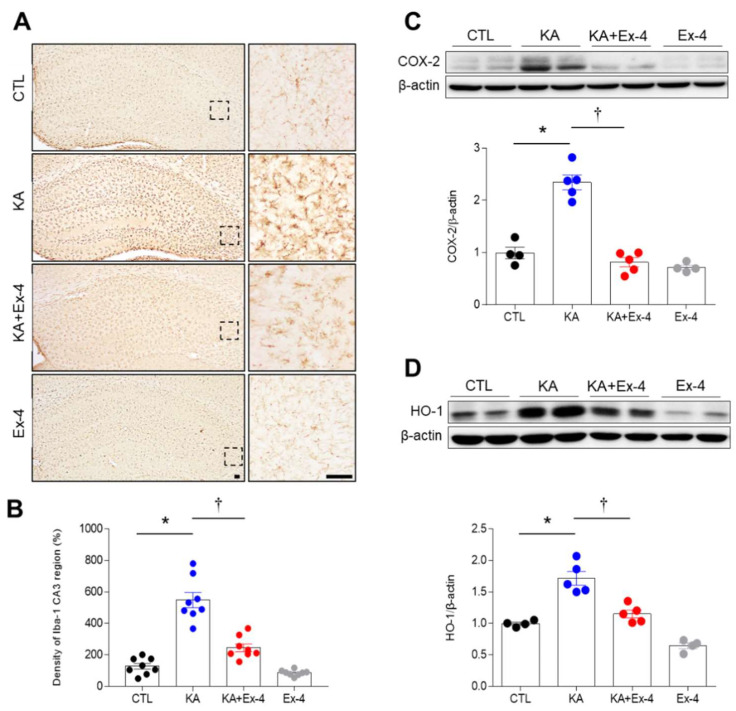
Effects of Ex-4 pretreatment on neuroinflammation in the hippocampus of KA-treated mice. (**A**) Representative images of Iba-1 immunohistochemistry are shown in the hippocampal CA3 region 24 h after KA treatment (*n* = 3–4 mice per group). The areas in dotted squares in the left panel are magnified on the right panel. Scale bar = 50 μm. (**B**) Quantitative result of Iba-1 positive cells are shown in 8-10 CA3 regions (*n* = 3–4 mice per group). (**C**,**D**) Western blot analysis of hippocampal COX-2 (**C**) and HO-1 (**D**) protein expressions. Densitometric values for COX-2 and HO-1 were normalized to β-actin levels (*n* = 4–5 mice per group). Data are presented as mean ± SEM. * *p* < 0.05 vs. CTL, † *p* < 0.05 vs. KA. The one-way ANOVA was used to detect any statistically significant differences between groups (**B**–**D**). B; *p* values = 0.0001 (KA), 0.0001 (KA + Ex-4), F (3, 28) = 52.26, COX-2/β–actin; *p* values = 0.0001 (KA), 0.0001 (KA + Ex-4), F (3, 14) = 52.88, HO-1/β–actin; *p* values = 0.0001 (KA), 0.0003 (KA+Ex-4), F (3, 14) = 36.74.

KA treatment remarkably increased expression of microglia marker Iba-1 24 h after injection, which was significantly attenuated by Ex-4 (Figure 4A). The Iba-1 immunoreactivity was intense in the CA3 region of KA-treated mice, whereas it was dramatically lower in KA + Ex-4-treated mice (Figure 4B). Finally, Ex-4 pretreatment led to a reduction in the protein expression of cyclooxygenase-2 (COX-2) and HO-1 increased by KA according to Western blot analyses (Figure 4C,D). These data suggest that Ex-4 pretreatment inhibited KA-induced inflammatory responses.

### 3.5. Ex-4 Pretreatment Attenuates Neuroinflammation in KA-Treated Mice

**Figure 5:**
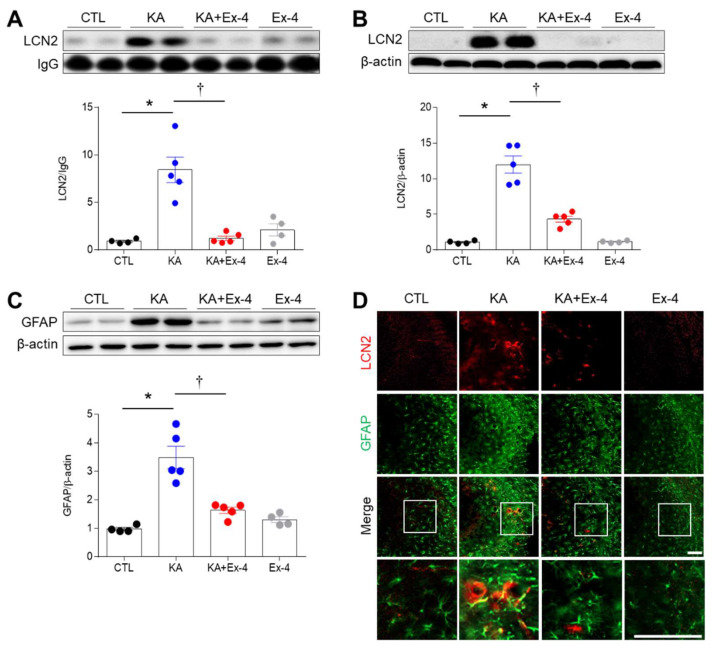
Effects of Ex-4 pretreatment on circulating and hippocampal LCN2 proteins in KA-treated mice. (**A**) Western blot showing circulation LCN2 levels. Ig G is used as an internal control to normalize protein levels (*n* = 4–5 mice per group). (**B**,**C**) Western blot analysis of LCN2 (**B**) and GFAP (**C**) expressions in the mouse hippocampus. Densitometric values for LCN2 and GFAP were normalized to β-actin levels (*n* = 4–5 mice per group). (**D**) Representative images of double immunofluorescence showing the co-localization of LCN2 and GFAP in the hippocampal CA3 region 24 h after KA treatment. The areas in white squares are magnified on the lowest panel. Scale bar = 50 μm. Data are presented as mean ± SEM. * *p* < 0.05 vs. CTL, † *p* < 0.05 vs. KA. The one-way ANOVA was used to determine whether statistically significant differences between groups (**A**–**C**). LCN2/IgG; *p* values = 0.0001 (KA), 0.0001 (KA + Ex-4), F (3, 14) = 20.02, LCN2/β–actin; *p* values = 0.0001 (KA), 0.0001 (KA + Ex-4), F (3, 14) = 53.09, GFAP/β–actin; *p* values = 0.0001 (KA), 0.0002 (KA + Ex-4), F (3, 14) = 23.88.

We next investigated expression levels of the acute pro-inflammatory cytokine, LCN2. Western blot analyses revealed that KA treatment significantly increased both circulating and hippocampal LCN2 expression levels. Critically, Ex-4 pretreatment significantly reduced these expression levels compared to KA treatment alone (Figure 5A,B). The increased GFAP protein in KA-treated hippocampus was decreased by Ex-4 pretreatment (Figure 5C). Finally, immunohistochemistry experiments indicated that LCN2-positive cells co-localized with GFAP-positive astrocytes in the KA group (Figure 5D). However, Ex-4 pretreatment reduced the number of co-localizing cells. These results suggest that Ex-4 pretreatment inhibits the KA-mediated induction of LCN2 expression in the hippocampus.

### 3.6. Ex-4 Pretreatment Protects against BBB Leakage in KA-Treated Mouse Hippocampus

**Figure 6:**
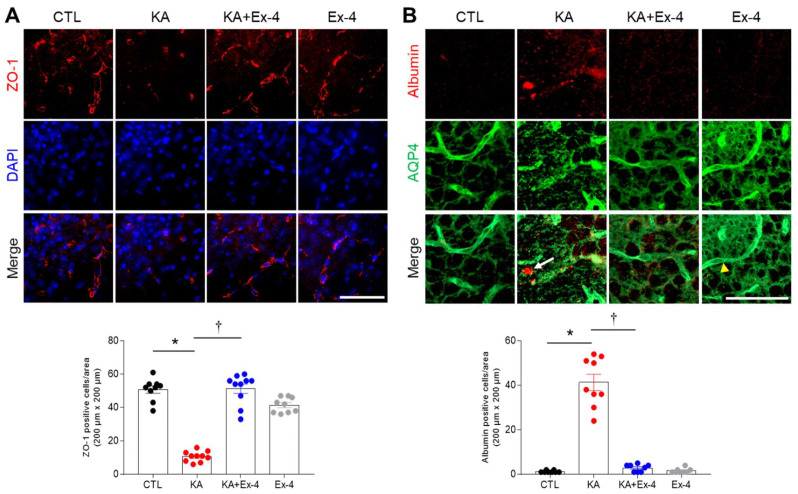
Effect of Ex-4 pretreatment on BBB leakage in KA-treated mouse hippocampus. (**A**) Representative images of immunofluorescence showing ZO-1 in the hippocampal CA3 region 24 h after KA treatment. Quantitative result of ZO-1 positive cells are shown in 8-10 CA3 regions (*n* = 3–4 mice per group). Nuclei were stained with DAPI. (**B**) Representative images of double immunofluorescence showing the albumin and AQP4 in the hippocampus 24 h after KA treatment. Quantitative results of albumin-positive cells are shown in 8–10 CA3 regions (*n* = 3–4 mice per group). White arrow and yellow arrow head indicate extravascular albumin and blood vessels, respectively. Scale bar = 50 μm. Data (*n* = 3–4 mice per group) are presented as mean ± SEM. * *p* < 0.05 vs. CTL, † *p* < 0.05 vs. KA. The one-way ANOVA was used to detect any statistically significant differences between groups (**A**,**B**). A; *p* values = 0.0001 (KA), 0.0001 (KA + Ex-4), F (3, 34) = 90.11, B; *p* values = 0.0001 (KA), 0.0001 (KA + Ex-4), F (3, 29) = 99.39.

We verified that KA induces BBB leakage by performing immunohistochemistry analyses of the tight junction protein ZO-1, serum albumin to mark BBB permeability, and the glial water-channel AQP4. KA-treated mice had significantly fewer ZO-1-positive cells. Intriguingly, the number of ZO-1-positive cells in mice treated with both Ex-4 and KA was similar to the numbers observed in control mice (Figure 6A). Additionally, we detected albumin expression near AQP4-positive astrocytes in the KA-treated hippocampus. We did not observe albumin near astrocytes in mice receiving the Ex-4 pretreatment (Figure 6B). These findings indicate that Ex-4 pretreatment protected against hippocampal BBB leakage in KA-treated mice.

### 3.7. Ex-4 Pretreatment Affects Hippocampal p-CREB Expression in KA-Treated Mice

**Figure 7:**
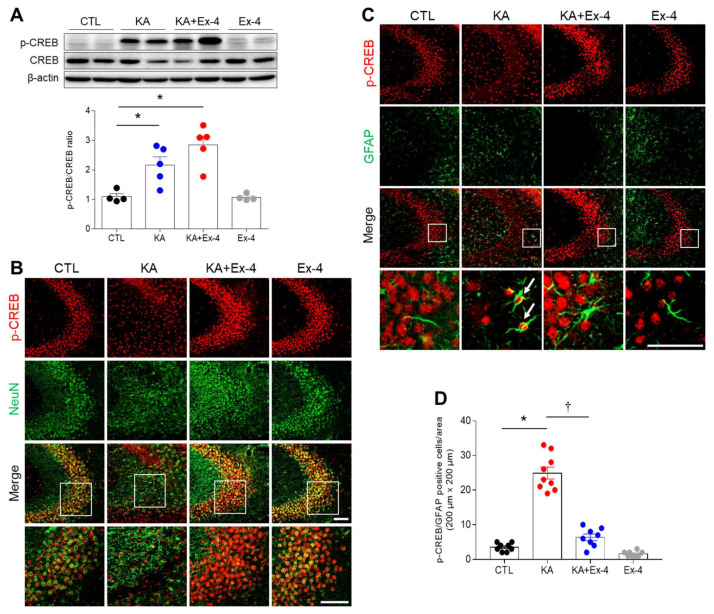
Effect of Ex-4 pretreatment on hippocampal p-CREB expression in KA-treated mice. (**A**) Western blot analysis of p-CREB expression in the mouse hippocampus 24 h after KA treatment. Densitometric values for p-CREB/CREB were normalized to β-actin levels (*n* = 4–5 mice per group). (**B**) Representative images of double immunofluorescence showing the p-CREB and NeuN in the hippocampus 24 h after KA treatment. The areas in white squares are magnified on the lowest panel. (**C**) Representative images of double immunofluorescence showing the p-CREB and GFAP. The areas in white squares are magnified on the lowest panel. Scale bar = 50 μm. (**D**) Quantitative result of co-localization of p-CREB and GFAP are shown in 8–10 CA3 regions (*n* = 3–4 mice per group). Data are presented as mean ± SEM. * *p* < 0.05 vs. CTL, † *p* < 0.05 vs. KA. The one-way ANOVA was used to detect any statistically significant differences between groups (**A**,**D**). p-CREB/CREB; *p* values = 0.0299 (KA), 0.0007 (KA + Ex-4), F (3, 14) = 13.54, D; *p* values = 0.0001 (KA), 0.0001 (KA + Ex-4), F (3, 29) = 104.6.

The p-CREB pathway plays a critical role in the process of cellular survival following neuronal injury [17]. Western blot analyses indicated that KA treatment induced phosphorylation of CREB. Furthermore, it appeared that the induction protein expression of p-CREB was stronger in mice than that received both KA and Ex-4 treatment. (Figure 7A). p-CREB-positive cells were co-localized with NeuN, a neuron marker, and co-localized with the astrocyte marker GFAP in the hippocampal CA3 region (Figure 7B,C). Notably, KA treatment reduced p-CREB levels in neurons and increased p-CREB in astrocytes, while Ex-4 pretreatment appeared to decrease levels of p-CREB in astrocytes. These results suggest that the activation of GLP-1R signaling by Ex-4-pretreatment upregulated p-CREB signaling and led to protection against hippocampal neuronal death in KA-treated mice.

## 4. Discussion

In this study, we investigated the possible neuroprotective effects of Ex-4 in mice with KA-induced seizures. Ex-4 pretreatment led to a decrease in markers of seizure activity, neuronal apoptosis, neuroinflammation, and BBB leakage in KA-induced seizure mice. In addition, increased circulating and hippocampal LCN2 protein levels in KA-treated mice were reversed by Ex-4 pretreatment. Furthermore, our results indicate that Ex-4 treatment activated GLP-1R/CREB signaling, which plays a critical role in protecting against excitotoxicity-induced neuronal damage. Therefore, we propose that Ex-4 pretreatment may exert a neuroprotective effect on seizure-related neurological disorders.

GLP-1/GLP-1R signaling regulates the protein expression of Bcl-2 family members (e.g., Bax, Bcl-2). These proteins tightly regulate the intrinsic apoptosis pathway, which is triggered by the mitochondrial release of cytochrome c and ultimately leads to caspase activation [27,28]. Active form of GLP-1: GLP-1 (7–36) protects hippocampal neurons against various insults including amyloid β, glutamate, oxidative stress, and endoplasmic reticulum stress [29]. In particular, Ex-4 pretreatment in models of myocardial infarction and subarachnoid hemorrhage downregulates apoptosis by inhibiting the release of cytochrome c via Bax/Bcl-2 regulation [9,30]. Liraglutide, an analog of GLP-1, contributes to the inhibition of the apoptotic pathway by upregulating anti-apoptotic factors (e.g., Bcl-2) and downregulating pro-apoptotic factors (e.g., Bax) and truncated caspase-3 [31]. Liraglutide affects neuroplasticity through mammalian target of rapamycin complex 1 (mTORC1) and α-Amino-3-hydroxy-5-methyl-4-isoxazolepropionic acid (AMPA) receptors and enhanced neurite outgrowth and synaptic density under dexamethasone-induced toxic conditions [32]. In addition, liraglutide was also shown to increase SH-SY5Y proliferation and p-CREB in traumatic brain injury [33]. Our results indicate that Ex-4 pretreatment in a KA-induced seizure model inhibited neuronal apoptosis by reducing the Bax/Bcl-2 ratio and inhibiting the release of cytochrome c. Together, these results demonstrate that pretreatments targeting the GLP-1/GLP-1R pathway, including Ex-4, may be used to reduce neuronal apoptosis in KA-treated mice.

In models of cerebral ischemia, intranasal administration of Ex-4 reversed the upregulation of caspase-3 in the hippocampus. Silencing expression of GLP-1R using short hairpin RNA reversed the effect of Ex-4 on cell survival [20]. In a diabetic retinopathy model, reduced GLP-1R expression increases reactive oxygen species (ROS), endoplasmic reticulum (ER), stress-mediated p53 and Bax promoter activity [34]. This apoptosis in retinal pigment cells was attenuated by Ex-4 treatment. Similarly, in the present study, GLP-1R expression decreased time-dependently after injection with KA (Figure 1B). We speculate that the reduction in GLP-1R protein levels was due to neuronal death. Our results indicate that Ex-4 pretreatment may have exerted an anti-apoptotic effect, which protected against KA-induced neuronal damage.

As shown in Supplementary Figure S1A, there was a significant increase in Ex-4 in the serum for animals treated with Ex-4. However, Ex-4 expression levels in the hippocampus were only slightly increased in the treatment groups compared to control animals. The results from ELISA were consistent with Western blot data (Supplementary Figure S1B). These data indicate that endocytic Ex-4 targets intracellular lysosomes and can be broken down. In particular, the ligand of the G-protein coupled receptors (GPCRs) is degraded in the endosome [35]. Primarily, Ex-4 stimulates GLP-1R, a type of GPCRs, and then promotes lysosome targeting [36]. We hypothesize that because Ex-4 is degraded in intracellular lysosomes in hippocampal cells, Ex-4 expression may not be detectable in the hippocampus by western blot or ELISA. Despite the lack of detection of Ex-4 in hippocampal cells in Ex-4 treated mice, our results indicate that Ex-4 inhibited KA-mediated reduction of GLP-1R protein. Furthermore, results from the Duolink assay indicated that close interactions of Ex-4 and GLP-1R proteins were enhanced by Ex-4 pretreatment in the hippocampus. Therefore, these findings suggest that Ex-4 can exert a neuroprotective effect through the pre-activation of GLP-1R.

Seizures cause hippocampal neuronal cell death and reactive gliosis, which produces pro-inflammatory cytokines [37,38]. Ex-4 has a neuroprotective effect in neuroinflammation via limiting oxidative damage and activated microglia [16]. Our study demonstrated that KA treatment induces reactive gliosis, as evidenced by the expressions of COX-2 and HO-1. These factors also induce an inflammatory response in the hippocampus. In accordance with our previous study [1], serum and hippocampal LCN2 proteins were significantly increased in KA-treated mice. Astrocytic LCN2 induced by cerebral ischemia or KA treatment promotes neuronal death, neuroinflammation, and oxidative stress [1,39]. These findings indicate that secreted LCN2 from damaged hippocampal lesions is significantly increased in KA-treated mice due to BBB leakage. Thus, we propose that Ex-4 pretreatment can protect against KA-induced LCN2 expression and BBB breakdown.

Inflammation is a key factor in the progression of BBB leakage, which results in brain injuries such as seizures and ischemic stroke [40,41]. BBB promotes a compartmentalized brain environment by limiting the diffusion of various molecules in the serum [42]. Because downregulation of ZO-1 appears to be the first stage in tight junction dysfunction leading to BBB permeability [43], and because albumin diffuses from the serum into the brain during BBB leakage [44], ZO-1 and albumin are reliable indicators of BBB leakage. We confirmed that BBB leakage occurred due to excitotoxicity caused by KA, resulting in the destruction of tight junction bonds in the hippocampus and albumin diffusion into the brain. The glial water channel AQP4 regulates water transport against an osmotic gradient involved in neural activity. Consistent with the present findings, our previous study showed that KA treatment increases the number of AQP4-positive astrocytes in the hippocampus [40]. In another study, AQP4 mRNA and protein levels in the hippocampus were significantly upregulated after eclampsia-like seizures [45]. Ex-4 attenuated all symptoms of BBB leakage, indicating that Ex-4 stabilized KA-induced BBB breakdown.

Ex-4 pretreatment activates the GLP-1R/CREB signaling pathway to exert neuroprotective effects [46,47]. According to previous studies, activation of p-CREB regulates the anti-apoptotic protein Bcl-2 to protect against apoptotic cell death after focal ischemia [48]. Furthermore, cerebral ischemia causes strong phosphorylation of CREB in neurons [17]. Our previous study demonstrated that cilostazol pretreatment decreases seizure activity and apoptotic neuronal cell death and increases CREB phosphorylation in the hippocampus [49]. p-CREB is mainly expressed in neurons and is known to be important for neuronal development, synaptic plasticity, and cell survival [16,50,51]. As shown in Figure 3B, there was an increase in hippocampal GLP-1R protein in KA+Ex-4-treated mice compared to only EX-4-treated mice. A Duolink assay also revealed that the close interaction of Ex-4 and GLP-1R in the hippocampus of KA + Ex-4-treated mice is higher than that of Ex-4-treated mice (Figure 3E). These findings imply that Ex-4 pretreatment has no effect on p-CREB expression in the hippocampus and do not activate the GLP-1R/CREB signaling pathway in unstressed conditions. However, CREB has been known as a key mediator of physiologic responses to seizures [52]. CREB phosphorylation is increased by seizure activity [53]. Therefore, we hypothesize that after Ex-4-induced GLP-1R activation, KA-induced GLP-1R/CREB signaling pathways protect hippocampal neurons from seizure activity, and that this positive feedback stimulation can enhance upregulation of GLP-1R/p-CREB protein. In accordance with our previous study [49], hippocampal p-CREB was also increased in KA-treated mice, but we saw no quantitative difference in p-CREB levels between KA-treated and KA+Ex-4-treated mice. Notably, although numbers of NeuN-positive p-CREB neurons were reduced in KA-treated mice, KA injection significantly increased the number of GFAP-positive p-CREB astrocytes. Hippocampal astrocytes were positive for p-CREB expression in intracerebroventricular KA-injected mice [54]. However, astrocytic p-CREB expression in the KA-treated hippocampus was not increased by Ex-4 pretreatment. Taken together, these findings suggest that Ex-4 pretreatment could activate GLP-1R/CREB signaling and provide protection against excitotoxicity-induced neuronal death.

## 5. Conclusions

In conclusion, our findings show that pretreatment of Ex-4 stimulated GLP-1R/CREB signaling pathway to protect against brain damage including hippocampal cell death, BBB leakage, neuroinflammation, and oxidative stress in mice with KA-induced seizures. Therefore, these data indicate that Ex-4 may have beneficial therapeutic effects on seizure activity and other brain injuries.

## Data Availability

The data presented in this study are available on request from the corresponding author.

## Supplementary Materials

The following are available online at https://www.mdpi.com/article/10.3390/cells10102527/s1, Table S1: List of primary antibodies. Figure S1: Ex-4 concentrations in serum and the mouse hippocampus 24 h after KA treatment, Figure S2: Effect of Ex-4 pretreatment on hippocampal GLP-1R and GFAP expressions in KA-treated mice.
