# Supplementary material for: Exendin-4 Pretreatment Attenuates Kainic Acid-Induced Hippocampal Neuronal Death
Source: Cells. 2021 Sep 24;10(10):2527. doi: 10.3390/cells10102527 (PMC8534217; doi:10.3390/cells10102527)
Supplement: Supplementary file 1 [file cells-10-02527-s001.zip › cells-1366785-SI.pdf]

cells

MDPI

Supplementary Material

Exendin-4 Pretreatment Attenuates Kainic Acid-Induced Hippocampal Neuronal Death

Yu-Jeong Ahn [1], Hyun-Joo Shin [1], Eun-Ae Jeong [1], Hyeong-Seok An [1], Jong-Youl Lee [1], Hye-Min Jang [1], Kyung-Eun Kim [1], Jaewoong Lee [1], Meong-Cheol Shin [2] and Gu-Seob Roh [1,*]

[1] Bio Anti-aging Medical Research Center, Department of Anatomy and Convergence Medical Science, Institute of Health Sciences, College of Medicine, Gyeongsang National University, Jinju 52727, Korea; ahnujung@naver.com (Y.-J.A.); k4900@hanmail.net (H.-J.S.); jeasky44@naver.com (E.-A.J.); gudtjr5287@hanmail.net (H.-S.A.); jyv7874v@naver.com (J.-Y.L.); gpals759@naver.com (H.-M.J.); kkejws@hanmail.net (K.-E.K.); woongs1111@gmail.com (J.L.)

[2] Research Institute of Pharmaceutical Sciences, College of Pharmacy, Gyeongsang National University, Jinju 52828, Korea; shinmc@gnu.ac.kr

* Correspondence: anaroh@gnu.ac.kr; Tel.: +82-55-772-8035; Fax: +82-55-772-8039

Supplementary Table S1. List of primary antibodies.

Antibody	Company	Catalog No.	Dilution(s)	Applications	Source
Albumin	abcam	ab192603	1:200	DIF	Rabbit
AQP4	Santa Cruz	sc-9888	1:200	DIF	Goat
Bax	Santa Cruz	sc-7480	1:1000	WB	Mouse
Bcl-2	Santa Cruz	sc-492	1:1000	WB	Rabbit
COX-2	Santa Cruz	sc-1745	1:1000	WB	Goat
CREB	Santa Cruz	sc-186	1:1000	WB	Rabbit
Cytochrome c	Santa Cruz	sc-13560	1:1000	WB	Mouse
Ex-4	Bioss	Bs-4121R	1:1000, 1:50	WB	Rabbit
GFAP	Sigma-Aldrich	G3893	1:1000, 1:500	WB, DIF	Mouse
GLP-1R	Santa Cruz	sc-66911	1:1000	WB	Rabbit
GLP-1R	Santa Cruz	sc-390774	1:1000, 1:50	DIF	Mouse
HO-1	Enzo	ADI-SPA 895	1:1000	WB	Rabbit
Iba-1	Wako	019-19741	1:200	IHC	Rabbit
IgG	Cell signaling	#3420	1:10000	WB	Mouse
LCN2	R&D	AF3508	1:1000, 1:250	WB, DIF	Goat
p-CREB (Ser 133)	Santa Cruz	sc-7978	1:1000, 1:200	WB, DIF	Rabbit
VDAC-1	abcam	ab15895	1:3000	WB	Rabbit
ZO-1	Santa Cruz	sc-33725	1:200	IF	Rat
β-actin	Sigma-Aldrich	A5441	1:10000	WB	Mouse

DIF; Double immunofluorescence, WB; Western blot, IHC; Immunohistochemistry, IF; Immunofluorescence.

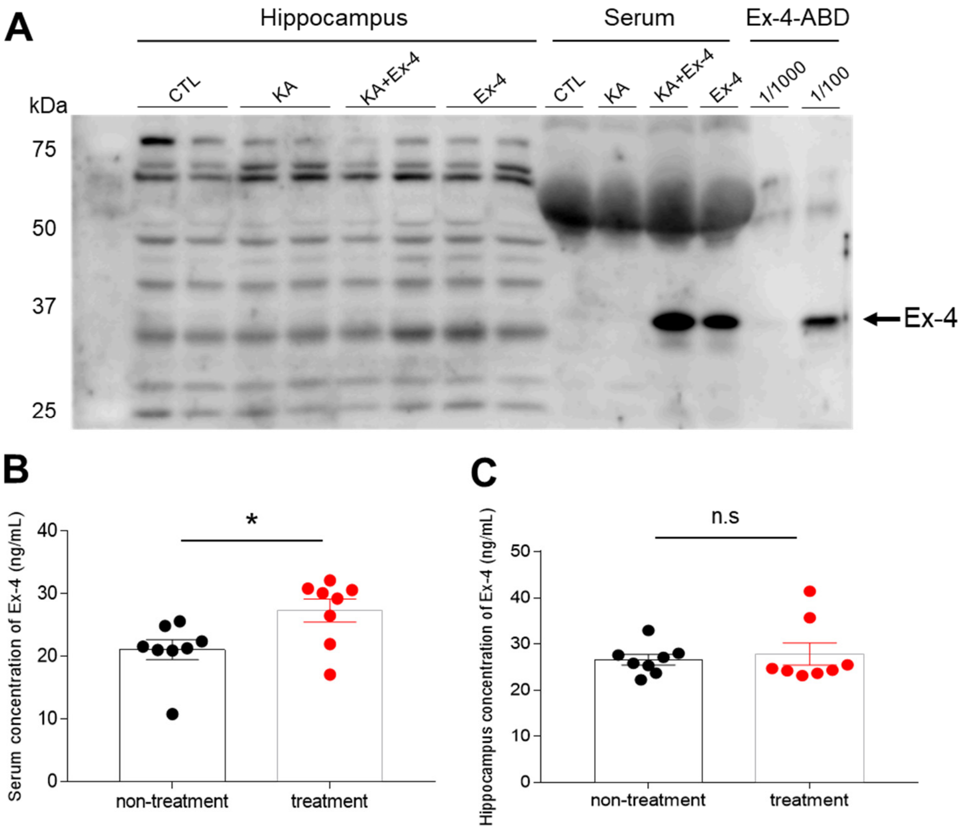

Supplementary Figure S1. Ex-4 concentrations in serum and the mouse hippocampus 24 h after KA treatment. (**A**) Western blot analysis of Ex-4 expressions in hippocampus, serum, and purified Ex-4-ABD dose-dependently expressions. (**B** and **C**) Ex-4 protein level from serum (**B**) and hippocampus (**C**) by using ELISA (n = 8 mice per group). n.s = not significant. Data are shown as mean ± SEM. * $p < 0.05$ vs. non-treated mice. The unpaired *t-test* was used to determine any statistically significant differences between groups (**B** and **C**). B; $p = 0.0228$, t = 2.556, df = 14, n = 8 mice per group.

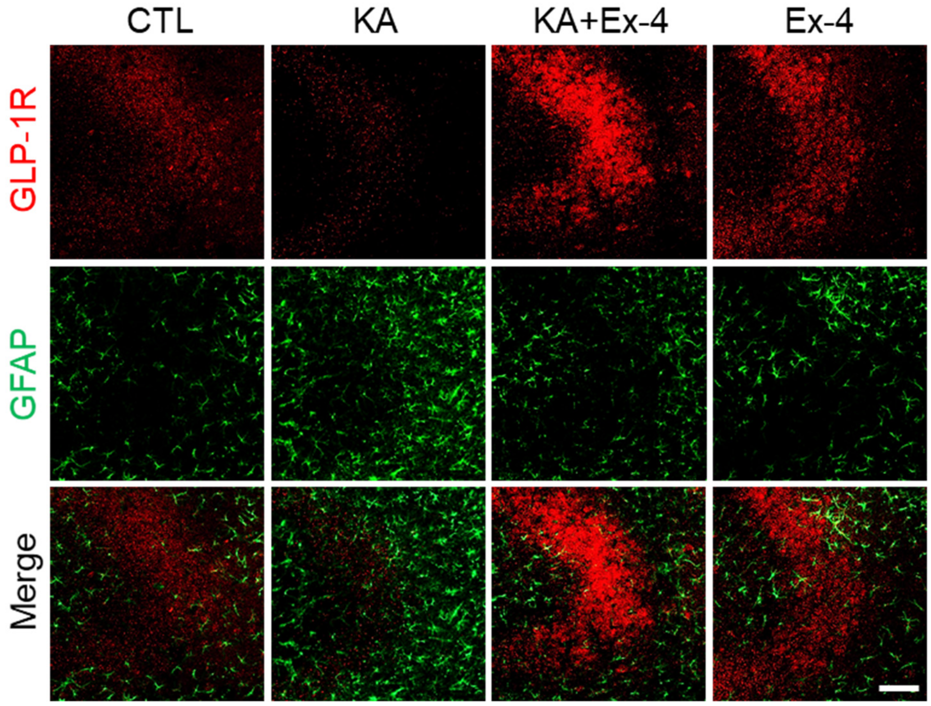

Supplementary Figure S2. Effect of Ex-4 pretreatment on hippocampal GLP-1R and GFAP expressions in KA-treated

mice. Representative images of double immunofluorescence showing the localization GLP-1R and GFAP in the hippocampal CA3 region. Scale bar = 50 μm.
